# Validity and Reproducibility of a Culture-Specific Food Frequency Questionnaire in Lebanon

**DOI:** 10.3390/nu12113316

**Published:** 2020-10-29

**Authors:** Raeda El Sayed Ahmad, Mariam Baroudi, Hibeh Shatila, Lara Nasreddine, Fatima Al Zahraa Chokor, Rana F. Chehab, Michele R. Forman, Farah Naja

**Affiliations:** 1 Department of Nutrition and Food Sciences, Faculty of Agriculture and Food Sciences, American University of Beirut, P.O. Box 11-0236, Riad El Solh, Beirut 11072020, Lebanon; rie08@mail.aub.edu (R.E.S.A.); mbb08@mail.aub.edu (M.B.); hs120@aub.edu.lb (H.S.); ln10@aub.edu.lb (L.N.); fc23@aub.edu.lb (F.A.Z.C.); 2 Department of Nutrition Science, Purdue University, West Lafayette, IN 47907, USA; rchehab@purdue.edu; 3 Nutrition Sciences, College of Health and Human Sciences, Purdue University, West Lafayette, IN 47907, USA; 4 Department of Clinical Nutrition and Dietetics, College of Health Sciences, Research Institute of Medical & Health Sciences (RIMHS), University of Sharjah, Sharjah 27272, UAE

**Keywords:** dietary assessment, food frequency questionnaire, validation, biomarkers, 24-h recall, triad methods, reliability, reproducibility, Lebanon

## Abstract

This study aims to assess the validity and reproducibility of a culture-specific semi-quantitative food frequency questionnaire (FFQ) for Lebanese adults. The 94-item FFQ captures intake of traditional Mediterranean dishes and Western food, reflective of current Lebanese nutrition transition. Among 107 participants (18–65 years), the FFQ was administered at baseline (FFQ-1) and one year thereafter (FFQ-2); 2–3 24-h recalls (24-HRs)/season were collected for a total of 8–12 over four seasons. A subset (*n* = 67) provided a fasting blood sample in the fall. Spearman-correlation coefficients, Bland–Altman plots, joint-classification and (ICC) were calculated. Mean intakes from FFQ-2 were higher than from the total 24-HRs. Correlations for diet from FFQ-2 and 24-HRs ranged from 0.17 for α-carotene to 0.65 for energy. Joint classification in the same/adjacent quartile ranged from 74.8% to 95%. FFQ-2-plasma carotenoid correlations ranged from 0.18 for lutein/zeaxanthin to 0.59 for β-carotene. Intra-class correlations for FFQ-1 and FFQ-2 ranged from 0.36 for β-cryptoxanthin to 0.85 for energy. 24-HRs carotenoid intake varied by season; combining season-specific 24-HRs proximal to biospecimen collection to the FFQ-2 improved diet-biochemical correlations. By applying dietary data from two tools with biomarkers taking into consideration seasonal variation, we report a valid, reproducible Lebanese FFQ for use in diet-disease research.

## 1. Introduction

Non-communicable diseases (NCDs) are a public health challenge worldwide, especially in low- and middle-income countries (LMICs) where more than three-quarters of NCD deaths occur [1]. Among environmental and lifestyle factors, diet plays a major role in the etiology and management of NCDs [2,3]. The last few decades have witnessed a plethora of epidemiological and laboratory-based studies that explore the diet-disease association [4,5,6,7,8,9].

An integral element to diet-disease research is the quality of the dietary assessment tool. Examples of such tools include: 24-h recalls (24-HRs), dietary records (DRs), narrative diet histories and food frequency questionnaires (FFQs). Dietary data may be complemented by biochemical measurement of intake from blood and urine samples for example [10,11]. DRs and 24-HRs estimate acute dietary intake; however, time, literacy, logistics and economic constraints associated with these methods render them less suitable for large-scale population-based studies of chronic intake [12]. The FFQ is less burdensome and costly and can be administered to large cohorts [12]. Furthermore, FFQ data can be aggregated to compare average nutrient intake of different groups, rank them and estimate absolute levels of nutrient and dietary intake over a relatively long period of the life course, ranging from months to years [12]. The latter feature of the FFQ is critical for the examination of the associations between diet and chronic diseases, such as NCDs [13]. As such, the FFQ is the most common tool in chronic disease epidemiological studies [12,14].

The challenge of documenting and comparing diverse dietary habits within and across cultures necessitates the development and use of valid and reproducible FFQs in culture-specific population research [15] to ensure accurate estimation of dietary intake. Alternatively, FFQs that are not validated may lead to systematic biases and random errors and obscure any diet-disease association [10,11,16]. Usually, a dietary assessment method is validated by comparing it to a superior method referred to as a “gold standard”, ideally with independent sources of measurement errors. Since few gold standards exist, reference methods like 24-HRs or DRs and biomarkers that are considered to have independent sources of errors are applied as comparators with FFQs in a triangulated fashion [12].

Over the past decade, FFQs were developed and validated among adults in countries of the Middle East and North African (MENA) region, such as the Islamic Republic of Iran, Palestine, the Kingdom of Saudi Arabia and Kuwait [17,18,19,20,21,22]. Lebanon, a small country in the Middle East, has long been known for its diverse cuisine and Mediterranean diet, which is rich in fruit, vegetables, whole grains and olive oil [23,24]. During the last few decades, however, Lebanon has experienced a nutrition transition with its traditional diet slowly eroding and being replaced by a more energy-dense, high-fat diet [23,25,26]. It is suggested that these changes in dietary intake could explain, in part, the epidemiological transition to increasing rates of NCDs in Lebanon [5,27,28]. Therefore, an eminent need exists to develop and validate a dietary assessment tool to accurately estimate usual dietary intake among adults in Lebanon. Such a tool should be able to capture the dietary intakes of traditional Mediterranean dishes and the more westernized energy-dense and high fat food items and validated by a dietary biomarker with independent measurement errors. Optimally, the dietary data would be collected over a year, with seasonal intake and biomarkers of intake as independent sources of error.

The aims of this study are to validate and to assess the reproducibility of a culture-specific semi-quantitative FFQ tailored for Lebanese adults using a triangulated approach. Specifically, the objectives of this study are to: (1) evaluate the relative validity of the FFQ in comparison with repeated 24-HRs and plasma biomarkers in a triangulated approach with a focus on carotenoid intake and their plasma concentrations taking into account seasonality of carotenoid intakes; (2) assess the reproducibility of the FFQ by administering two FFQs, once at baseline and the second 12 months later.

## 2. Materials and Methods

### 2.1. Study Participants

Adults aged 18 to 65 years (*N* = 120) were recruited at the American University of Beirut (AUB) between December 2016 and March 2017. In the Lebanese private sector, AUB is the largest employer in Lebanon with 3500 non-academic employees and over 800 faculty members across socio-economic groups [29,30]. Flyers were distributed around campus and verbal announcements were made in department meetings. Interested individuals contacted the research team by email or telephone. Subjects were first screened for the following eligibility criteria: full or part time employment at AUB, of Lebanese nationality or a resident of Lebanon for more than ten years, conversant in Arabic or English language, not pregnant or breastfeeding and free of any chronic health condition that requires dietary modifications. On the AUB campus, a large number of visitors come daily from many parts of the country. In order to ensure the continuity of enrollment in the study and for a clearer definition of the study sample, the criteria ‘full or part time employment at AUB’ was added. The study protocol was approved by the Institutional Review Board of the Social and Behavioral Sciences at AUB under the protocol number (NUT.FN.22). Prior to participation, all subjects provided a signed informed consent.

### 2.2. Study Design

**Figure 1:**
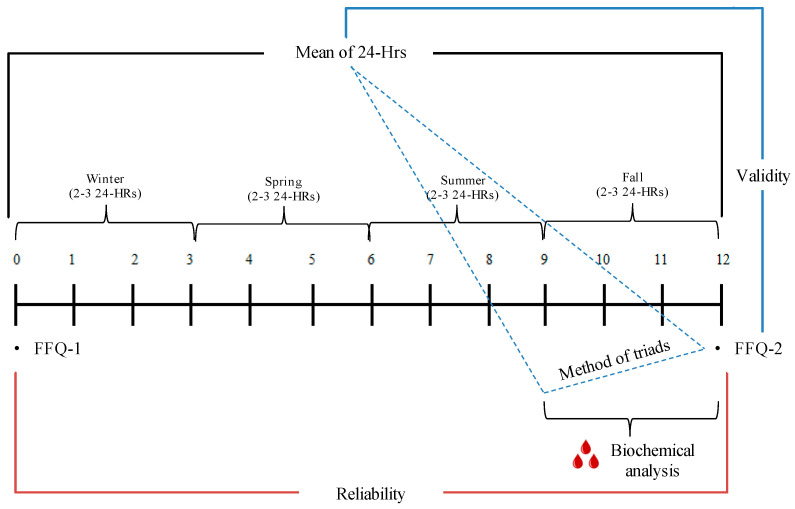
Design of the validation and reliability study of a culture specific food frequency questionnaire (FFQ) among Lebanese adults.

The study was designed as a two-point-in-time administration of an FFQ, once at baseline (FFQ-1) and then at the end of one year (FFQ-2) (Figure 1), coupled with two to three 24-HRs that were collected on one to two weekdays and one weekend day during each of four seasons for a total of 8 to 12 24-HRs. In addition to the FFQ-1 at baseline, participants completed a socio-demographic and lifestyle questionnaire and anthropometric measurements were recorded. A fasting blood sample for biochemical analysis was collected in the fall concurrent with 24-HRs.

### 2.3. Data Collcetion

Data collection occurred between December 2016 and March 2018. Throughout the study period, participants were encouraged to maintain their regular dietary habits. All interviews were completed by the same trained research dietitian. Data collection encompassed three forms of nutritional assessment, notably dietary, anthropometric and biochemical assessment, plus dietary tool development and a sociodemographic-lifestyle questionnaire. Given nutrition transition and the seasonality of fruit and vegetable intake, the focus was on carotenoid and vitamin A intakes and their individual and total plasma concentrations.

### 2.4. Dietary Assessment: Food Frequency Questionnaire (FFQ): Development and Collection

A 94-item semi-quantitative FFQ was developed to assess usual dietary intake among Lebanese adults over the past year. The questionnaire had three sections: the food list, the portion size, and the frequency of intake. The following steps were taken to compile the food list. First, 24-HRs from a previously conducted national survey were reviewed to identify foods that are commonly consumed in Lebanon. The survey was conducted in the year 2009 with a total number of adult participants of 2680 [31]. Food items which were frequently cited (>5%) were included in the food list of the FFQ. The review process continued until saturation occurred and no new foods were identified. The total number of 24-HRs reviewed was 200. Second, a panel of experts reviewed this food list for its clarity and presentation. The panel included two licensed dietitians, a public health nutritionist and a nutrition epidemiologist. The review of the food list was carried out in iterations until a consensus was achieved on the inclusion/exclusion of food items. The format of the questionnaire was vetted by members of the panel who had extensive experience in administering surveys and were familiar with the participants’ reactions and preferences in this regard. Third, this food list was then examined by 10 AUB employees for its comprehensiveness and cultural specificity. These employees were selected to be similar in characteristics to the target population. Finally, the list was compared to previously published FFQs validated in neighboring countries since a Lebanese FFQ for adults had not yet been reported [22,32,33]. The final FFQ food list had a total of 94 items categorized in 23 food groups and an open-ended section in which participants were asked to provide information about regularly consumed foods or beverages that did not appear on the food list. The Arabic and English versions of this questionnaire can be obtained upon request from the authors.

As for portion size, subjects were given the option to report the portion size in terms of common household measurements (teaspoons, tablespoons and cups) or using a standard two-dimensional (2-D) food portion visual chart. The latter chart was developed by Nutrition Consulting Enterprises and validated among adults aged 20 to 70+ years as part of the Framingham Heart Study [34] to assist in quantifying portion size in dietary intake surveys.

Research dietitians asked the participants to refer to their diet during the year prior to the interview. Participants reported their frequency of intake by indicating how many times each food item was consumed per day, week or month or seldom/not at all. In addition, to account for seasonal variation, the intake of some food items, such as certain fruits, was reported per season and later adjusted to estimate the frequency of consumption over the year. The frequency of consumption in units of daily intake of each of the 94-item FFQ was multiplied by its reported portion size to calculate the total amount in grams of food intake per day [35]. The same FFQ was administered at baseline (FFQ-1) and one year later (FFQ-2) to assess the reproducibility of the FFQ. The average time to complete each FFQ was 30–35 min.

### 2.5. Dietary Assessment: 24-h Recalls (24-HRs) Using the Multiple Pass Food Recall Approach

During the one-year data collection period, a total of 8–12 24-HRs per participant were collected via face-to-face interviews. The interviews were conducted by a trained research dietitian in a private setting at the participant’s office or, if not available, at the Nutrition and Food Sciences department at AUB. The Multiple Pass Food Recall five-step approach, which was developed by the United States Department of Agriculture (USDA), was used to take into account and attenuate the limitations of the traditional 24-HRs [36,37]. For each 24-HR, the research dietitian obtained information about the time when the meal was consumed, the food consumed, its portion size, preparation method, and brand of the food/beverage/supplement. The two or three 24-HRs per season were collected on one or two weekdays and one weekend day. The participants were notified of the time of their upcoming 24-HR interviews on the same day to ensure that they maintain their usual food intake. The Nutritionist Pro software (version 5.1.0, 2014, First Data Bank, Nutritionist Pro, Axxya Systems, San Bruno, CA, USA) was used to analyze the dietary intake data from the FFQ and the 24-HRs. Lebanese composite dishes were added to the software database using standardized recipes of single food items from the USDA database.

### 2.6. Blood Sample Collection and Laboratory Analysis

During the fall either proximal to or one season prior to the FFQ-2 but at the same time as the fall 24-HRs, a 5 mL fasting blood sample was collected from consenting participants. Blood samples were centrifuged and stored in aliquots at <−80 °C before shipping on dry ice to Craft Technologies Inc. Laboratories (Wilson, NC, USA) for analysis. Blood samples were analyzed for levels of carotenoids, retinols, as well as the lipid profile (total cholesterol, HDL-C and triglycerides). The compounds were identified and quantified using high performance liquid chromatography (HPLC). For serum extraction and HPLC methods, a modification of the procedures described in De Roos et al. [38] was used. In-house quality control (QC) samples were analyzed at the beginning, end and at 24 batched-sample intervals. Linear calibration curves were prepared consisting of multiple concentrations of analytes which spanned the physiological levels of the analyzed carotenoids in serum. The calibrants included lutein, zeaxanthin, α- and β-cryptoxanthin, lycopene and α- and β-carotene. Serum quantification was performed by internal standard calibration using peak area ratios. The relative standard deviation of analytes in QC samples ranged from 3 to 10% between batches. Plasma cholesterol was measured by an enzymatic spectrophotometric technique using a Vitros 350 analyzer, with a % coefficient of variation below 2% (Ortho-Clinical Diagnostics, Johnson and Johnson, 50–100 Holmers Farm Way, High Wycombe, Buckinghamshire HP12 4DP, UK).

### 2.7. Baseline Anthropometric Assessment and Socio-Demographic and Lifestyle Questionnaire

During the first visit, trained dietitians measured the weight and height of the participants using SECA 877 scales for measurement of weight to the nearest 0.1 kg. The scales were regularly calibrated using known weights (2, 4 and 6 kg); if any of the readings was 0.1 kg below or above the known weight, the scale was returned to the manufacturer for professional calibration. Height (cm) was measured using SECA 213 stadiometer to the nearest 0.1 cm. Participants were measured barefoot and with light clothing. Body mass index (BMI) was calculated as weight (kg)/height^2^ (m^2^). All measurements were taken twice and the average of the two readings was used in the study. If any pair of readings differed by more than 0.1 kg for weight or 0.7 cm for height (7 mm), the trained dietitians measured and recorded a second and, if necessary, a third pair of readings [39]. All trained dietitians received extensive training before the start of data collection. Remedial training was conducted as required.

The socio-demographic and lifestyle questionnaire included information about the participant’s age (in years), sex, place of residence (Beirut, outside Beirut), marital status (single—including widowed and divorced; married), educational level (up to intermediate level, high school and university/technical diploma), type of employment (academic; non-academic), monthly income (<LBP 3 million; ≥LBP 3 million) and crowding index (<1 versus ≥1 individual/room). Crowding index is calculated as the ratio of the number of household residents to the number of rooms in the house and it has been used as a proxy for socio-economic status in Lebanon [40,41,42,43]. In addition, information regarding smoking and physical activity was collected. Subjects were classified as either non-smokers—including past smokers—or current smokers. For smokers, participants were asked about the average number of cigarettes smoked per day and the duration of smoking. The Arabic version of the International Physical Activity Questionnaire (IPAQ) [44,45] was administered to assess the participants’ physical activity level (low, moderate, high) using a reference period of the week prior to the interview.

### 2.8. Sample Size for the Diet-Diet Comparison or Diet-Biochemical Comparison

Of the 120 recruited participants, 110 completed the dietary study (dropout rate: 8.3%). This sample size is within the range (100–200) suggested by Willett and Lenhart (1998) for a validation study of dietary questionnaires and is higher than the minimum sample size for such studies (100) [13,46]. The main reasons for attrition were: unable to complete a minimum of two 24-HRs per season or left AUB during the study. After removing outliers (*n* = 3) based on energy intake using boxplots, a total of 107 participants (*n* = 65 men; 60.7%) were included in the diet-diet analysis. The sample for the diet-biochemical data analysis had 67 individuals who consented for blood collection from among the 107 in the diet-diet analysis. It remains important to note that, using the Goldberg method, 87 subjects (79.1%) were classified as plausible reporters and 23 subjects (20.9%) as under-reporters [47].

### 2.9. Statistical Analysis

Socio-demographic and anthropometric characteristics of the participants were described using frequencies with percentages (%) and means with standard deviations (SD) for categorical and continuous data, respectively. Nutrient intakes estimated from FFQ-2 and 24-HRs were energy-adjusted using the residual method [12]. To assess FFQ validity, dietary intakes derived from FFQ-2 were compared to the mean of the 8–12 24-HRs during the year before the FFQ-2 in the form of a mean difference. Paired t-tests were computed to compare season-specific average dietary intake of carotenoids. Furthermore, Spearman rank correlation coefficients (r) between intakes from FFQ-2 and the mean of the total 24-HRs were calculated. In addition, the Bland–Altman approach [48] was used to graphically assess and visually compare the agreement between the two dietary tools. Specifically, the difference in intake from the FFQ-2 and mean 24-HRs was plotted against the mean intake estimated by the two measurements ((FFQ-2+ mean 24-HRs)/2). The plots included lines for the mean difference and the Limits of Agreement (LOA), defined as mean difference ± 1.96 × SD.

To assess diet-biochemical validity, Spearman rank correlation coefficients were calculated to examine the association of nutrient intakes (carotenoids and retinols) estimated from the 24-HRs and FFQ-2 with their corresponding plasma biomarker levels. Since most carotenoids are transported by plasma lipoproteins, plasma concentrations of carotenoids were adjusted for plasma cholesterol concentrations, in addition to energy. Further adjustments were made for BMI and smoking status since they are recognized covariates of carotenoid intake and concentrations [49,50] We conducted an additional sensitivity analysis of the diet-biochemical correlations formulated as a take-off to the approach of Freedman L et al. [51], in which the average of the 24-HRs collected proximal to the blood collection are then averaged with the FFQ-2 to estimate the averaged carotenoid intake from the 24-HRs + FFQ-2 and then correlated with individual and total carotenoids.

To assess FFQ reproducibility, intraclass correlation coefficients (ICC) were calculated to examine the agreement (between FFQ-1 and FFQ-2) to rank individuals according to their energy, macronutrient and carotenoid/retinol intake. The correlation coefficients (Spearman and ICC) were calculated using log-transformed data since most nutrient distributions were skewed. We also calculated weighted kappa (κw) and the joint classification of a nutrient in the same and adjacent quartiles multiplied by 100 percent to estimate the percent agreement. Values of κw between 0.40 and 0.59 were considered moderate, 0.60 to 0.79 substantial and 0.8 outstanding, as per Landis and Koch [52]. The Statistical Package for Social Sciences 24.0 (SPSS for Windows, 2013, SPSS Inc., Chicago, IL, USA) was used for data analysis. A *p*-value < 0.05 was considered statistically significant.

## 3. Results

Socio-demographic and lifestyle characteristics, including the BMI, of the study sample for the diet-diet analysis and then a similar description of the diet-biochemical participants compared with those who did not participate are presented in Table 1.

### 3.1. Diet-Diet FFQ Validity

Table 2 presents the mean ±SD and Spearman’s correlation coefficients for energy, macronutrients, fiber and cholesterol in addition to the carotenoid/tocopherol/retinol intake estimated from FFQ-2 and the average of the 8–12 24-HRs. Mean intakes estimated by FFQ-2 were higher than those reported by the 24-HRs for most macronutrients and lower for vitamin A, α-carotene, β-carotene, Lutein+Zeaxanthin, lycopene and the total carotenoids. Spearman correlation coefficients ranged from 0.17 for α-carotene to 0.65 for energy, with most correlations significant with the exception of α-carotene and lycopene.

**Figure 2:**
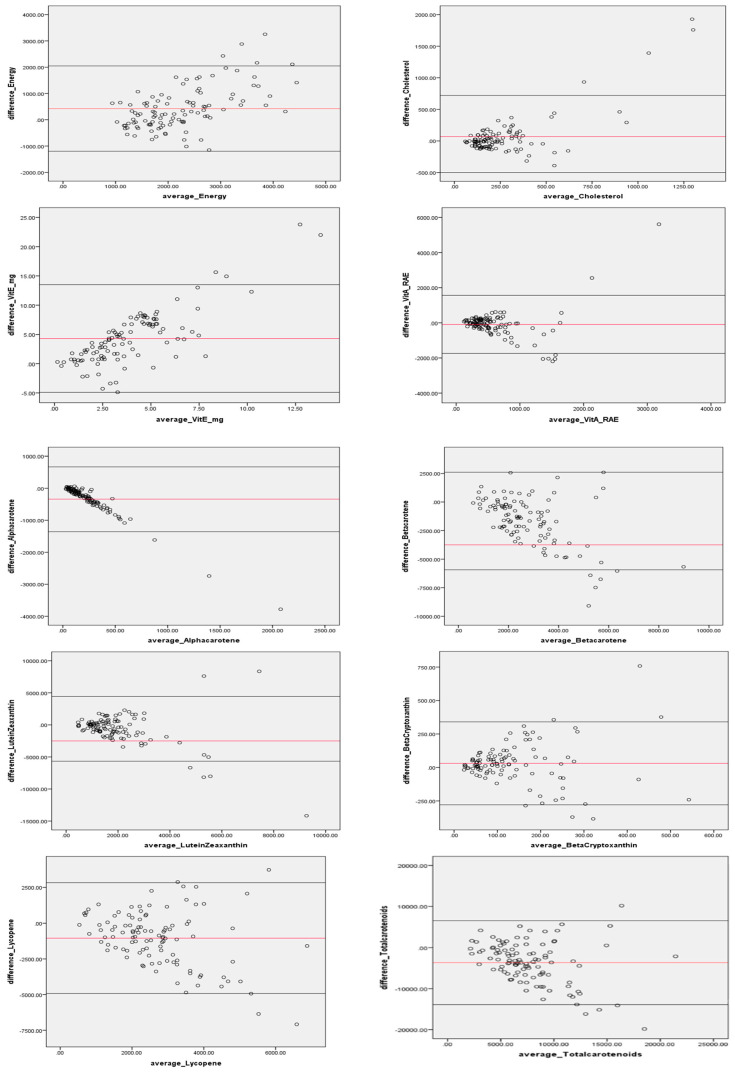
Bland–Altman plots for energy and select nutrients (*n* = 107).

The Bland–Altman plots revealed the comparability of dietary intake estimated from FFQ-2 and from the 24-HRs (Figure 2). The mean difference in energy and cholesterol values increased with increasing intakes, with R2 of 0.36 and 0.57, respectively, and most values within the 95% LOA. The upward trend in the regression lines for these two dietary components indicated that a greater mean difference between estimates from the FFQ-2 and the mean of the 24 HRs was observed with increasing intakes. In contrast, α and β-carotene, Lutein+Zeaxanthin and lycopene revealed lower, not necessarily significant, R2 and a negative regression line, indicating a trend toward higher FFQ-2 estimates with lower mean 24-HRs.

**Figure 3:**
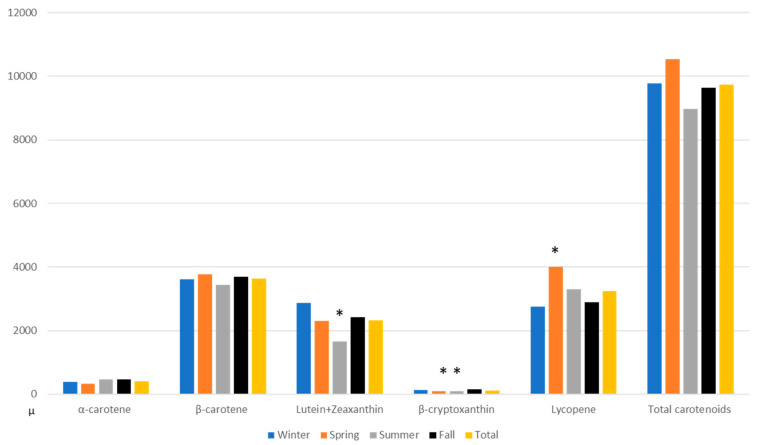
Using data from 24-HRs, comparison of fall intake of carotenoids (μg) with intakes during winter, spring and summer as well as with average of year round intakes (*n* = 107). * The *p*-values for the comparisons are derived from a paired t-test.

Since carotenoids are concentrated in fruit and vegetables with seasonal differences due to access and availability apparent in many LMICs (Forman MR et al., 1999), we examined whether mean season-specific dietary intake of carotenoids varied. In Figure 3, carotenoid-specific seasonal intake (as derived from 24-HRs) varied specifically: Lutein+Zeaxanthin intake varied between the summer and fall, β-cryptoxanthin intake varied between the spring and fall as well as the summer and fall; lycopene intake varied between the spring and the fall. In an analysis of the seasonal intake of the macronutrients, energy intake was higher in winter, summer and fall than in spring.

The diet–plasma correlations appear in Table 3. After adjusting for energy, cholesterol, BMI and smoking status, the Spearman rank correlation coefficients between plasma biomarkers and dietary intake estimated by the FFQ-2 were significant (*p* < 0.05) for α- and β-carotene, Lutein/Zeaxanthin and β-cryptoxanthin. The correlation coefficients based on the averaged 24-HRs across the seasons were significant for β-cryptoxanthin and α- and β-carotene. Indeed, significant correlation coefficients were observed for β-carotene and β-cryptoxanthin regardless of the dietary tools. In a sensitivity analysis [51] (Table 4), we observed correlations ranging from 0.30 to 0.50 based on the averaged 24-HRs for the three days in fall when blood was collected combined with the average intake from the FFQ-2 and plasma biomarker concentrations. These diet–biochemical correlations were higher for α- and β-carotene and lycopene than the correlations based on the fall-only 24-HRs or the FFQ-2.

### 3.2. FFQ Reproducibility

The reproducibility of the FFQ was assessed by calculating the ICC, the weighted kappa and the percent agreement within the same or adjacent quartiles of intake between FFQ-1 and FFQ-2 (Table 5). The ICCs were significant for energy and all of the nutrients and ranged from 0.36 for Lutein+Zeaxanthin to 0.85 for energy. The weighted kappa values were highest for energy and macronutrient intake with the majority above 0.5. Lower weighted kappa values were observed for most micronutrients and ranged from 0.25 for Lutein+Zeaxanthin to 0.4 for vitamin A. Percent agreement in classifying subjects into the same or adjacent quartile ranged from 74.8% for Lutein+Zeaxanthin to 95.3% for energy and saturated fat.

## 4. Discussion

Among 107 adults aged 18–65 years who were employed at AUB, we examined the validity and reliability of a newly-developed FFQ specific to the Lebanese population by comparing nutrient intakes from two FFQs, FFQ1 at the beginning and FFQ-2 at the end of a year, with those estimated from a total of 8–12 24-HRs collected over four seasons of the same year. We further evaluated the validity of the FFQ by examining the correlation between dietary intakes of and plasma concentrations of carotenoids, and retinol. Nutrient intake, but not for carotenoid and retinol, estimated from the FFQ-2 was higher than from the 24-HRs; rather, the carotenoid and retinol intakes from the 24-HRs were higher than from the FFQ-2. The Spearman rank correlations for dietary intake from the FFQ with those from the 24-HRs were generally high for energy and most macronutrients but not the carotenoids. Bland–Altman plots corroborated diet–diet correlations and comparisons. The FFQ–plasma carotenoid correlations were stronger than those for intake from the 24-HRs; correlations were consistently strong for β-carotene and β-cryptoxanthin regardless of the dietary tool. Seasonal intake of each carotenoid varied as did the peak season of intake for each individual carotenoid. After taking into account seasonal intake of the carotenoids based on the 24-HRs proximal (in the fall) to FFQ-2 plus intake from FFQ-2, recalculated correlations were improved for lycopene and α-carotene and remained significant for the other carotenoids. The reproducibility of the FFQ by comparing dietary intake assessed by the FFQs administered at the beginning and at the end of the study revealed high ICCs and high concordance/joint classifications except for lycopene and for vitamin A. Thus, we approached FFQ validation through a triangulated approach, i.e., the method of the triads—using two dietary tools, one of which offered seasonal lens to assess variation in carotenoid intake and plasma carotenoid concentrations—to capture relatively independent measures for comparison to assess the validity of a culture-specific FFQ for Lebanon.

### 4.1. FFQ Validity: Diet-Diet and Diet-Biochemical Comparisons

The FFQ overestimated dietary intake of most of the nutrients compared to the 24-HRs, similar to other studies [33,53,54,55,56,57,58]. The 24-HRs overestimated dietary intake of carotenoids and vitamin A compared to the estimates from the FFQ-2. In contrast, prior research used different modes such as dietary records or ASA24 rather than 24-HRs for comparison [57]. Several FFQ validation studies reported correlation coefficients between the tested FFQ and 24-HRs similar to ours [17,33,59]. Comparing coefficients between studies is challenging given the variation in study populations, study design, nutrient, dietary tools and statistical analysis.

The reasons for the difference in estimated carotenoid intake from the FFQ-2 and 24-HRs may be due to the seasonal availability and accessibility of fruit and vegetables rich in carotenoids in LMICs. Indeed, individual carotenoid intakes from the averaged 24-HRs varied by season. In a sensitivity analysis combining the 24-HR data from the fall season of the blood collection with FFQ-2, we reported a range of significant diet–biochemical correlations from 0.30 to 0.50 for individual carotenoids and higher correlations for two carotenoids than those correlations based on the FFQ-2 alone, while no associations were noted between dietary intake and vitamin A, even after considering the seasonal variation. Such a finding could be due to the fact that this vitamin is found in particular foods, which the food list of the FFQ may have not addressed in particular but rather were listed in aggregate format with other fruits and vegetables. Such foods include carrots, broccoli, cantaloupe and squash. Future studies are encouraged to consider separating these foods items, in the case that the focus is vitamin A intake, its seasonal variation and association with blood biomarker. That said, our approach of combining 24-HR dietary data simultaneous with the timing of the blood draw with long-term intake estimates from the FFQ provides a different lens on the application of dietary assessments to understanding seasonal intakes due to access and availability and offers an alternative to the estimation of diet–biochemical carotenoid correlations that may underpin diet–disease associations.

The results of the Bland–Altman plots confirmed the overestimation of dietary intake by the FFQ for macronutrients, similar to previous studies [60]. The Bland–Altman plots also confirmed that the FFQ underestimated intake compared to the 24-HRs for total and individual carotenoids and vitamin A. Thus, the two approaches from correlations and Bland–Altman plots supported the observation that estimated intakes of macronutrients are higher from the FFQ-2 than the 24 HRs while estimated micronutrients are lower from the FFQ-2 than the 24 HRs, and the sensitivity analysis revealed improved carotenoid correlation using both dietary tools. Further, we checked the outliers in the Bland–Altman plots for different nutrients and found different participants, not the same, for each nutrient, indicating no systematic bias for nutrient intakes amongst individuals.

### 4.2. FFQ Validity: Diet-biochemical Correlations

The diet–plasma carotenoid correlations were significant, ranging from 0.3 to 0.6. Our results are in accord with several studies. In an assessment among 3110 Australian participants of [61] a 121-item FFQ developed for The Melbourne Collaborative Cohort Study, Pearson diet–plasma carotenoid correlation coefficients were lowest for lycopene and β-carotene at 0.28 (95% CI: 0.24, 0.31), followed by Lutein/Zeaxanthin at 0.29 (95% CI: 0.26, 0.32) and α-carotene at 0.40 (95% CI: 0.37, 0.43), and highest for β-cryptoxanthin at 0.46 (95% CI: 0.43, 0.49) after adjustment for energy intake and plasma cholesterol level. The adjusted correlation coefficient for retinol was 0.037 (0.02, 0.08), lower than that of all carotenoids. The correlation between intake of and plasma level of vitamin A was not significantly similar to another study by [62], potentially because plasma retinol levels are largely under homeostatic control by the liver and not reflected by intake.

### 4.3. FFQ Reproducibility

The FFQ was administered once at baseline and then a second time, one year after the first administration, to the same subjects to examine reproducibility. The time interval between the administration of the two FFQs is an important factor that influences reproducibility [60]. We chose a time period of one year to avoid participants recalling their answers to FFQ-1 when completing FFQ-2, thus leading to an overestimation of the FFQ’s reproducibility. At the same time, we aimed to minimize potential temporal changes in subjects’ dietary intakes over a long period which would lead to an underestimation of the FFQ’s reproducibility.

The ICC coefficients from this study ranged from 0.44 to 0.85 for macronutrients and from 0.36 to 0.70 for micronutrients, comparable with most studies that assessed FFQs’ reproducibility [54,56,58]. The majority of coefficients were greater than 0.5, suggesting a high reproducibility of the FFQ according to [46] where the minimum correlation for an assessment tool to be considered reproducible is 0.5. Of note, two recent Lebanese FFQs reported higher ICCs but the FFQ administration was repeated within a much shorter interval of either one or four months and there were no biochemical assessments [63,64].

To further assess the FFQ’s reproducibility, the weighted kappa was calculated, which, unlike the ICC, gives a single value to represent agreement and adjusts for chance agreement and the degree of disagreement [65]. The weighted kappa showed moderate agreement for macronutrients (0.5–0.6) and from fair to moderate agreement for micronutrients (0.3–0.4) [65]. Of note, more than half of the nutrients had moderate agreement in FFQ-1 and FFQ-2. The weighted kappa values in a study by [54] were higher for protein, total fat and cholesterol but comparable for the rest of the assessed nutrients. Our FFQ correctly classified participants into the same and adjacent quartiles, suggesting a high degree of agreement between FFQ-1 and FFQ-2. The nutrients that had exceptionally poor agreement between the FFQ and the mean of 24-HRs and the two administrations of the FFQ in the present study, mainly vitamin A, α-carotene and β-carotene, were not consistently reported in other validation studies [54,55,56,66].

### 4.4. Limitations and Strengths

Our study has strengths and limitations. The study was designed to capture intake from the FFQ and seasonal intake from 2–3 24-HRs, providing the opportunity to address variation in intake and evaluate whether seasonal intake influences diet–biochemical correlations. The study had a large sample size to collect diet, but the reduced subset for biochemical analysis may have limited our capacity to detect associations. The one-year duration of the study was essential to capture seasonality and is in line with the duration of many FFQs [12,13] given the need to examine long-term intake and disease relations. Besides the reduced sample for biochemical analysis, we recognize that the sampling frame did not lead to a representative sample of the overall Lebanese population; rather, the sample reflected AUB membership, the largest employer in Lebanon. Future studies are needed to examine the validity of the FFQ among adults of various age ranges.

## 5. Conclusions

In an assessment of the relative validity and reproducibility of a 94-item FFQ for Lebanese adults, we report its validity using the method of triads. Recognizably being validated in the same population, it was intended to be used as it renders the greatest chance for valid and reliable findings [10,67].

The results of this study support that the FFQ is an adequate method for dietary assessment among adult Lebanon population. The use of this FFQ will assist in the assessment of adult Lebanese dietary intake in epidemiological studies and will aid in the understanding of the diet–disease link [16]. In brief, the findings of this study suggest that this FFQ is valid and reproducible in ranking the energy and most nutrient intakes of Lebanese adults, with a caveat related to seasonality of intake of carotenoid-rich foods for which 24-HRs proximal to the FFQ could enhance correlations in this population.

## Figures and Tables

**Table 1:** Baseline characteristics for study participants and for those who did (Yes) or did not (No) provide a blood sample.

Baseline Characteristic ^a^	Total (*N =* 107)	Provided Blood Sample		*p*-Value ^c^
		Yes (*n* = 67)	No (*n* = 40)	
Age (years) ^b^	38.4 ± 0.9	38.5 ± 1.1	38.3 ± 9.5	0.899
Male	65(60.7)	50(74.6)	15(37.5)	0.000
Female	42(39.2)	17(25.4)	25(62.5)	
Residents outside Beirut	59(55.1)	43(64.2)	16(40.0)	0.015
Married	60 (56.1)	41(61.2)	19(47.5)	0.167
Education level				
Up to intermediate level	24(22.4)	18(26.9)	6(15.0)	0.209
High school	14(13.1)	10(14.9)	4(10.0)	
University/technical diploma	69(64.5)	39(58.2)	30(75.0)	
Non-academic employment	91(85.0)	59(88.1)	32(80.0)	0.258
Monthly income ≥USD 2000	55(51.4)	29(43.3)	26(65.0)	0.030
Crowding index ≥1 individual/room	56(52.3)	41(61.2)	15(37.5)	0.018
Current smoker	58(54.2)	43(64.2)	15(37.5)	0.007
Physical activity level				
Low	26(24.3)	14(20.9)	12(30.0)	0.432
Moderate	69(64.5)	44(65.7)	25(62.5)	
High	12(11.2)	9(13.4)	3(7.5)	
Body Mass Index (kg/m^2^)				
Normal (18.5–24.9)	29(27.1)	8(11.9)	21(52.5)	0.000
Overweight (25.0–29.9)	42(39.3)	34(50.7)	8(20.0)	
Obese (≥30)	36(33.6)	25(37.3)	11(27.5)	
Number of 24-HRs completed during the study				
8	4(3.7)	2(3.0)	2(5.0)	0.678
9	17(15.9)	9(13.4)	8(20.0)	
10	23(21.5)	16(23.9)	7(17.5)	
11	48(44.9)	32(47.8)	16(40.0)	
12	15(14.0)	8(11.9)	7(17.5)	

24-HRs: 24 h recalls; ^a^ Values presented as *n* (%) unless otherwise stated. ^b^ Values presented as mean ± SD. ^c^ *p*-value derived using chi-square test for categorical variables and independent t-test for continuous variables and compares characteristics of participants who provided blood samples to those who did not.

**Table 2:** Mean nutrient intake estimated by FFQ-2 and by all 24-HRs and Spearman correlation coefficients for FFQ-2 vs. 24-HRs (*N* = 107).

Nutrient	FFQ-2	24-HRs	FFQ-2 vs. 24-HRs
	Mean ± SD ^a^	Mean ± SD ^a^	Spearman’s r ^b^
Energy (kcal)	2538.5 ± 1065.5	1992.7 ± 612.5	0.65 **
Proteins (g)	93.5 ± 51.8	74.7 ± 26.8	0.34 **
Carbohydrate (g)	280.4 ± 132.9	216.9 ± 74.5	0.44 **
Fiber, Total (g)	23.6 ± 11.0	18.3 ± 6.7	0.55 **
Fat, Total (g)	113.2 ± 52.1	90.1 ± 29.5	0.45 **
Cholesterol (mg)	305.1 ± 363.5	236.5 ± 148.3	0.44 **
Vitamin A (RAE)	564.8 ± 670.6	655.7 ± 568.8	0.29 *
α-tocopherol (mg)	14.3 ± 7.4	10.2 ± 4.4	0.52 **
α-carotene (μg)	77.3 ± 47.3	415.9 ± 510.4	0.17
β-carotene (μg)	1963.5 ± 1221.5	3630.6 ± 2196.2	0.29 *
Lutein+Zeaxanthin	1703.9 ± 1469.3	2318.6 ± 2246.0	0.24 *
β-cryptoxanthin	151.0 ± 127.6	120.3 ± 123.8	0.23 *
Lycopene	2194.3 ± 1278.8	3240.8 ± 1837.7	0.18
Total carotenoids	6068.8 ± 3554.2	9726.1 ± 3554.2	0.22 *

RAE: Retinol Activity Equivalents; r: correlation coefficients; SD: Standard deviation; 24-HRs: 24 h recalls; FFQ-1: 1st food frequency questionnaire collected (baseline). ^a^ Mean values are log-transformed. ^b^ Spearman correlation coefficients are energy-adjusted. * *p*-value < 0.05; ** *p*-value < 0.01.

**Table 3:** Spearman correlation coefficients for diet–plasma carotenoids and vitamin A with specific intake estimated from 24-HRs and with FFQ-2 (*n* = 67).

Nutrient	Plasma Biomarkers (µg/mL)	24-HRs (µg) ⁱ	FFQ-2 (µg) ⁱ	Plasma Biomarkers vs. 24-HRs		Plasma Biomarkers vs. FFQ-2	
	Mean ± SD			r ^1^	r ^2^	r ^1^	r ^2^
α-carotene	0.07 ± 0.04	368.10 ± 520.76	83.78 ± 54.46	0.23	0.37 **	0.35 **	0.38 **
β-carotene ^b^	0.32 ± 0.13	3415.57 ± 2064.03	2128.01 ± 1390.64	0.39 **	0.46 **	0.55 **	0.59 **
Lutein+Zeaxanthin	0.40 ± 0.14	2024.59 ± 1384.79	1925.19 ± 1734.62	0.22	0.18	0.40 **	0.34 **
β-cryptoxanthin	0.13 ± 0.09	108.54 ± 115.73	163.94 ±146.34	0.41 **	0.36 **	0.37 **	0.29 *
Lycopene ^a^	0.38 ± 0.12	3178.56 ± 1838.48	2382.33 ± 1499.60	0.15	0.13	0.21	0.24
Vitamin A ^d^	0.58 ± 0.16	680.39 ± 605.29	653.60 ± 822.29	−0.09	−0.09	0.04	0.15

r: correlation coefficients; SD: Standard Deviation; 24-HRs: 24 h recalls; FFQ-2: 2nd food frequency questionnaire collected (one year after baseline). ⁱ All nutrients derived from the 24-HRs and FFQ are in µg, except Vitamin A (RAE). ^a^ Plasma total lycopene was calculated as the sum of t-lycopene+c-lycopene. ^b^ Plasma total β-carotene was calculated as t-β-carotene+c-β-carotene. ^d^ Plasma vitamin A was considered as retinol. ^1^ Correlation coefficients after log-transforming and energy-adjusting nutrient intakes and cholesterol-adjusting carotenoids. ^2^ Correlation coefficients after log-transforming and energy-adjusting nutrient intakes, cholesterol-adjusting carotenoids and adjusting for BMI (Body Mass Index) and smoking status. * *p*-value < 0.05; ** *p*-value < 0.01.

**Table 4:** Sensitivity analysis (*n* = 67). Mean carotenoid intakes by average fall 24-HRs and FFQ-2 and nutrient plasma concentration and Spearman correlation coefficients for subjects who have undergone blood tests (*n* = 67).

Nutrient	Plasma Biomarker (µg/mL)	Fall 24-HRs	Fall 24-HRs and FFQ-2 ⁱ	Plasma Biomarkers vs. Fall 24-HRs		Plasma Biomarkers vs. Fall 24-HRs and FFQ-2	
	Mean ± SD			r ^1^	r ^2^	r ^1^	r ^2^
α-carotene	0.07 ± 0.04	368.73 ± 931.58	226.25 ± 475.53	0.29 *	0.27 *	0.31 *	0.46 **
β-carotene ^b^	0.32 ± 0.13	3364.09 ± 3303.89	2746.05 ± 1991.15	0.25 *	0.26 *	0.42 **	0.47 **
Lutein+Zeaxanthin	0.40 ± 0.14	2208.02 ± 2880.18	2066.61 ± 1797.02	0.24	0.19	0.37 **	0.30 *
β-cryptoxanthin	0.13 ± 0.09	119.91 ± 201.83	141.93 ± 139.78	0.31 *	0.27 *	0.38 **	0.27 *
Lycopene ^a^	0.38 ± 0.12	2597.29 ± 2739.25	2489.82 ± 1765.30	0.16	0.18	0.25 *	0.32 **
Vitamin A ^d^	0.58 ± 0.16	789.62 ± 1475.13	721.61 ± 845.18	−0.16	−0.09	−0.01	0.04

r: correlation coefficients; SD: Standard Deviation; 24-HRs: 24 h recalls; FFQ-2: 2nd food frequency questionnaire collected (one year after baseline). ⁱ All nutrients derived from the 24-HRs and FFQ are in µg, except Vitamin A (RAE). ^a^ Plasma total lycopene was calculated as the sum of t-lycopene+c-lycopene. ^b^ Plasma total β-carotene was calculated as t-β-carotene+c-β-carotene. ^d^ Plasma vitamin A was considered as retinol. ^1^ Correlation coefficients after log-transforming and energy-adjusting nutrient intakes and cholesterol-adjusting carotenoids. ^2^ Correlation coefficients after log-transforming and energy-adjusting nutrient intakes, cholesterol-adjusting carotenoids and adjusting for BMI and smoking status. * *p*-value < 0.05; ** *p*-value < 0.01.

**Table 5:** FFQ reproducibility: Intraclass correlation coefficients, weighted kappa and same and adjacent % agreement for comparison of FFQ-1 and FFQ-2 (*n* = 107).

Nutrients	FFQ-1	FFQ-2	FFQ-1 vs. FFQ-2		
	Mean ± SD	Mean ± SD	ICC ^#^	Weighted Kappa	Same and Adjacent % Agreement
Energy (kcal)	3061.8 ± 1479.4	2538.5 ± 1066.5	0.85 **	0.56	95.3
Protein (g)	112.2 ± 59.8	93.5 ± 51.8	0.44 *	0.56	92.5
Carbohydrate(g)	345.6 ± 178.0	280.4 ± 132.9	0.56 **	0.51	90.7
Fiber, Total(g)	27.4 ± 12.9	23.6 ± 11.0	0.64 **	0.50	85.1
Fat, Total(g)	134.1 ± 67.4	113.2 ± 52.1	0.67 **	0.49	89.7
Cholesterol(mg)	345.4 ± 298.7	305.1 ± 363.5	0.55 **	0.46	85.1
α-Tocopherol(mg)	16.0 ± 8.6	14.3 ± 7.4	0.56 **	0.39	86.0
Vitamin A (RAE)	694.1 ± 786.9	564.8 ± 670.6	0.44 **	0.40	85.1
α-carotene(μg)	93.0 ± 60.7	77.3 ± 47.3	0.62 **	0.36	82.2
β-carotene(μg)	2328.4 ± 1333.4	1963.5 ± 1221.5	0.58 **	0.36	84.1
Lutein+Zeaxanthin	2116.1 ± 1762.7	1703.9 ± 1469.3	0.36 *	0.25	74.8
β-cryptoxanthin	210.8 ± 157.2	151.0 ± 127.6	0.70 **	0.38	80.4
Lycopene	3180.3 ± 3151.1	2194.3 ± 1287.8	0.63 **	0.32	82.2
Total Carotenoids	7861.0 ± 5135.3	6068.8 ± 3554.2	0.53 **	0.33	80.1

ICC: Intraclass correlation coefficients; SD: Standard Deviation; FFQ-1: 1st food frequency questionnaire collected (baseline); FFQ-2: 2nd food frequency questionnaire collected (one year after baseline). **^#^** ICCs are derived from log-transformed values that are energy-adjusted except for energy. * *p*-value < 0.05; ** *p*-value < 0.01.
